# Isolated hypoglossal nerve mononeuropathy associated with preeclampsia in late pregnancy: a case report

**DOI:** 10.1186/s13256-026-05935-x

**Published:** 2026-03-23

**Authors:** Sandeep Kaur, Akashdeep Singh Chauhan

**Affiliations:** 1 Department of Surgery and Cancer, Faculty of Medicine, Imperial College London, Hammersmith Campus, London, W12 0NN UK; 2 Indian Institute of Public Health-Delhi, Public Health Foundation of India, Gurugram, 122102 India

**Keywords:** Preeclampsia, Hypoglossal nerve mononeuropathy, Cranial mononeuropathy, Pregnancy complications, Neuroimaging, Proteinuria

## Abstract

**Background:**

Neurological complications in preeclampsia are uncommon and typically central in origin. Cranial mononeuropathies, especially involving the hypoglossal nerve, are exceedingly rare.

**Case presentation:**

We report a case of isolated hypoglossal nerve mononeuropathy in a 31-year-old, Indian, primigravida at 37 weeks of gestation with preeclampsia. The patient presented with mild dysphagia and dysarthria. Neurological examination revealed tongue deviation to the right, suggestive of left hypoglossal nerve mononeuropathy. Magnetic resonance imaging of the brain, including T1/T2-weighted sequences, fluid-attenuated inversion recovery, and diffusion-weighted imaging with apparent diffusion coefficient sequences, and magnetic resonance angiography, followed by contrast-enhanced magnetic resonance imaging of the neck and oral cavity, showed no infarct, hemorrhage, mass lesion, or compressive pathology. The patient was managed conservatively with blood pressure optimization, and symptoms resolved completely within 90 days postpartum.

**Conclusion:**

At least four cases of hypoglossal nerve mononeuropathy associated with preeclampsia have been reported in literature. This case adds to the emerging evidence that cranial mononeuropathies may represent a rare but important neurological manifestation of preeclampsia and highlights the need for urgent neuroimaging and careful blood pressure control in pregnant and/or postpartum patients presenting with bulbar symptoms.

## Clinical significance

Cranial mononeuropathies are rare but documented neurological complications of preeclampsia, most frequently affecting the facial and oculomotor nerves [1]. Involvement of the hypoglossal nerve is exceedingly uncommon. Brain magnetic resonance imaging (MRI) plays a pivotal role in excluding alternative etiologies such as neoplastic, vascular, or infectious causes. Management is centered on the optimization of blood pressure and the underlying obstetric condition. Neurological recovery is typically spontaneous and complete [1, 2].

## Background

Preeclampsia affects approximately 3–5% of pregnancies and typically presents after 20 weeks of gestation [3]. It is defined by new-onset hypertension (≥ 140/90 mmHg) with proteinuria (≥ 300 mg/24 hours) or, in the absence of proteinuria, the presence of end-organ dysfunction. Hemolysis, elevated liver enzymes, and low platelet count (HELLP) syndrome represents a severe variant with multisystem involvement [3].

## Case presentation

**Fig. 1:**
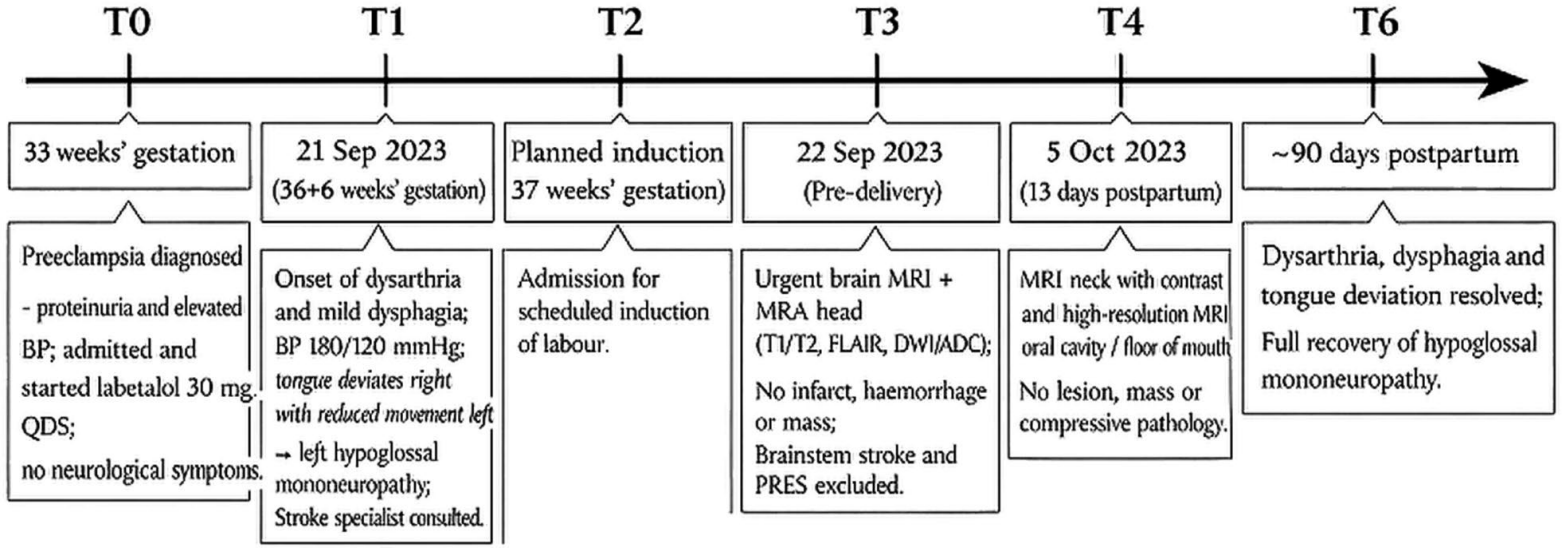
Timeline of clinical events

A 31-year-old, Indian, primigravida at 33 weeks’ gestation was admitted with a diagnosis of preeclampsia with proteinuria. Her antenatal course had been uneventful, with no significant past medical history. After a 1-week hospital stay, she was discharged on labetalol 30 mg four times daily (QDS) and scheduled for induction of labor at 37 weeks (Fig. 1).

**Fig. 2:**
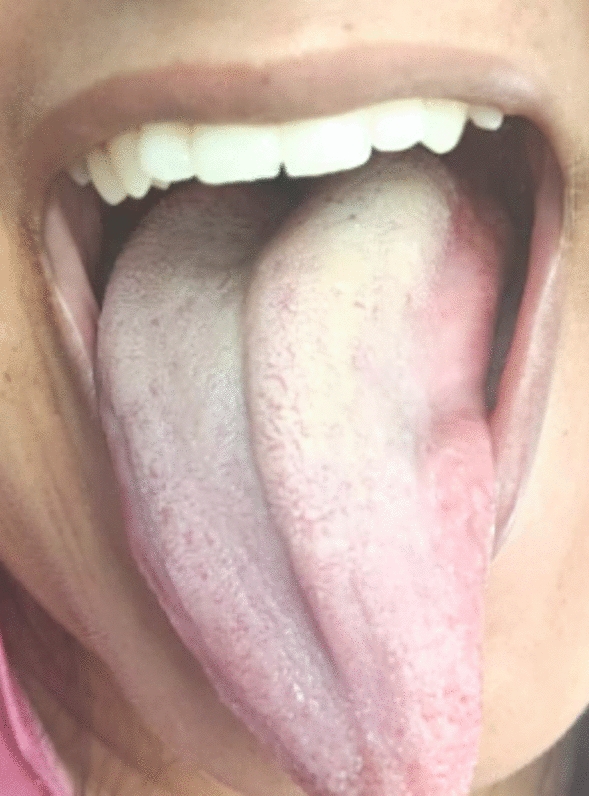
Deviated tongue of the patient with left hypoglossal nerve mononeuropathy

However, on 21 September 2023 (36 + 6 weeks gestation), 1 day prior to the planned induction, she returned with complaints of mild dysphagia and dysarthria. On examination, her blood pressure was markedly elevated at 180/120 mmHg. Neurological assessment revealed a normal-appearing tongue at rest, without atrophy or fasciculations; however, on protrusion, the tongue deviated to the right with reduced movement toward the left, suggestive of left hypoglossal nerve mononeuropathy (Fig. 2). Induction of labor was initiated that night, and she had a forceps-assisted vaginal delivery, with episiotomy and epidural analgesia, the following day.

Urgent magnetic resonance imaging (MRI) of the brain and magnetic resonance angiography (MRA) of the head were performed on 22 September 2023, while she was still pregnant. The MRI protocol included T1- and T2-weighted, fluid-attenuated inversion recovery (FLAIR), and diffusion-weighted imaging with apparent diffusion coefficient (ADC) maps. The neuroradiology report described a normal cerebrum, cerebellum, and brainstem, with no restricted diffusion to indicate acute infarction and no FLAIR abnormality to suggest posterior reversible encephalopathy syndrome. There was no susceptibility-related signal change to indicate hemorrhage or other blood products. Cerebrospinal fluid (CSF) spaces were symmetrical with no midline shift or hydrocephalus, and major arterial and venous flow voids were preserved, arguing against cerebral venous thrombosis, aneurysm, or arterial dissection. The skull base appeared normal.

The patient remained afebrile throughout. Her blood pressure was managed with atenolol and nifedipine, with readings stabilizing at 140/90 mmHg. Laboratory investigations showed mildly elevated liver transaminases. MRA ruled out vascular malformations. Imaging did not reveal signal changes or enhancement along the hypoglossal nerve pathway or in the brainstem.

In the early postpartum period, the patient underwent contrast-enhanced MRI of the neck and upper aerodigestive tract, along with high-resolution MRI of the oral cavity and floor of mouth (Fig. 1). These assessments demonstrated normal appearances of the upper aerodigestive tract, with uniform tongue signal without denervation changes or focal mass lesion; normal carotid spaces; and no lymphadenopathy, vascular malformation, or abnormal enhancement along the expected course of the hypoglossal nerve. No neoplasm or compressive lesion was identified in the neck, skull base, or floor of mouth. A diagnosis of isolated hypoglossal nerve mononeuropathy in the setting of preeclampsia was made.

Given the absence of a treatable structural lesion and the presence of an isolated, nonprogressive hypoglossal nerve mononeuropathy, a conservative management strategy was adopted. Treatment focused on the underlying preeclampsia, with optimization of blood pressure using labetalol and nifedipine, and standard obstetric management including induction of labor. In line with the local protocol for severe preeclampsia, the patient also received low-molecular-weight heparin (dalteparin) for venous thromboembolism prophylaxis during admission and for 1 week after discharge; this was given for thromboprophylaxis rather than as a specific treatment for the neuropathy. No nerve-directed therapy was initiated. The bulbar symptoms improved gradually and resolved completely within 90 days postpartum, in parallel with normalization of blood pressure.

## Discussion

Cranial mononeuropathies are a recognized but uncommon neurological complication of hypertensive disorders of pregnancy [4]. Facial nerve palsy is most frequently reported, followed by abducens nerve involvement. Hypoglossal nerve mononeuropathy in association with preeclampsia is extremely rare; at least four cases have been described in literature [1, 5–7]. Our case adds a further example of this rare association.

The pathophysiology of cranial nerve involvement in preeclampsia remains uncertain. Preeclampsia is characterized by systemic endothelial dysfunction, microangiopathy, and vasospasm, creating a hypercoagulable, vasospastic state that may compromise perfusion of cranial nerves via the vasa nervorum or small skull-base vessels and result in transient ischemic mononeuropathy [8]. This mechanism has been proposed for facial and abducens nerve palsies and in previously reported cases of hypoglossal nerve mononeuropathy associated with preeclampsia, which interpret the deficit as microvascular rather than structural, consistent with normal structural imaging [1, 5–7].

In addition, fluid retention and capillary leak may lead to perineural edema in confined spaces such as the hypoglossal canal, producing reversible compressive neuropathy, while hormonal and immunological changes in pregnancy may further destabilize endothelial function [9]. Although direct pathological evidence at the level of the hypoglossal nerve is lacking, and these mechanisms remain partly speculative, they are supported indirectly by the clinical pattern of lower motor neuron signs, normal central imaging, and complete postpartum recovery in our patient and in previously reported cases [6].

A comprehensive evaluation was undertaken to exclude alternative causes of hypoglossal nerve mononeuropathy. Brain MRI with diffusion-weighted imaging (DWI)/ADC, FLAIR, and MRA excluded brainstem infarction, hemorrhage, mass lesion, venous thrombosis, aneurysm, and carotid or vertebral artery dissection. Contrast-enhanced MRI of the neck and oral cavity/floor of mouth demonstrated normal tongue signal, normal carotid spaces, and no focal mass, lymphadenopathy, vascular malformation, or abnormal enhancement along the hypoglossal nerve course, arguing against skull-base, parapharyngeal, or carotid-space neoplasm and other compressive lesions. There was no history of head or neck trauma, recent surgery, or airway manipulation, and the patient remained afebrile with no clinical or laboratory evidence of infection. Neurological examination showed isolated hypoglossal involvement without other cranial nerve or long-tract signs. In this context, attribution of the hypoglossal nerve mononeuropathy to preeclampsia represents a diagnosis of exclusion.

The temporal pattern in this case supports a direct association between severe preeclampsia and hypoglossal nerve involvement. The mononeuropathy arose abruptly at 37 weeks’ gestation, coinciding with a hypertensive crisis in the setting of established preeclampsia, and resolved fully over the postpartum period as blood pressure and preeclampsia-related abnormalities improved. Management focused on tight blood pressure control, standard obstetric care, and venous thromboembolism prophylaxis, without nerve-specific therapy; cranial neuropathy improved in parallel with stabilization of the hypertensive/preeclamptic state. Although causality cannot be definitively proven, the close temporal coupling between disease activity and neurological deficit, together with the exclusion of other etiologies, favors a direct association rather than a coincidental occurrence.

Beyond optimized blood pressure control, magnesium sulfate where indicated, and postpartum thromboprophylaxis, there is currently no evidence-based disease-specific treatment for hypoglossal nerve mononeuropathy in this setting. Previously reported cases, as well as ours, have improved with supportive management alone once structural, vascular, infectious, and traumatic causes have been excluded.

## Conclusion

Isolated hypoglossal nerve mononeuropathy is an exceptionally rare complication of preeclampsia. In this case, onset during a hypertensive crisis, normal structural and vascular neuroimaging, exclusion of alternative etiologies, and complete postpartum recovery support a benign, reversible, preeclampsia-associated mononeuropathy. Clinicians should consider this diagnosis in pregnant or postpartum patients with new-onset dysarthria, dysphagia, or tongue deviation, and hypertension, and perform prompt neuroimaging while optimizing blood pressure and obstetric management.

## Data Availability

Given the sensitivity of the data, it will not be made publicly available. However, anonymized health records can be shared upon reasonable request.
